# A simple, anesthesia-free infusion technique for in vivo metabolic tracing

**DOI:** 10.1126/sciadv.aeg1157

**Published:** 2026-09-11

**Authors:** Yumi Kim, Samantha Caldwell, Meredith Long, Dominique Abrahams, Margi Baldwin, Gina M. DeNicola

**Affiliations:** ^1^ Department of Metabolism and Physiology, H. Lee Moffitt Cancer Center and Research Institute, Tampa, FL 33612, USA.; ^2^ Division of Comparative Medicine, University of South Florida, Tampa, FL 12901, USA.

## Abstract

Accurate metabolic flux analysis requires tracer delivery that preserves physiological metabolism. Current methods may distort metabolism through isoflurane anesthesia, surgical stress, or complex procedures. We demonstrate that isoflurane anesthesia profoundly alters serum and tissue metabolism across multiple pathways. In serum, acylcarnitines and fatty acids were broadly decreased, whereas amino acid metabolites and select nucleotide species were increased. Across multiple organs, isoflurane induced coordinated metabolic remodeling and distinct tissue-specific responses, including glycolytic remodeling in the brain and amino acid accumulation in the pancreas. To address these metabolic disturbances, we established a nonsurgical tail vein catheterization method completed in minutes under brief isoflurane anesthesia that enables multihour tracer infusion in awake, freely moving mice. Using U-^13^C_6_-cystine infusion, this method achieved robust cysteine labeling and downstream labeling comparable to jugular infusion while maintaining circulating cystine pools closer to physiological levels. This platform provides a practical approach for in vivo stable isotope tracing under more physiological conditions.

## INTRODUCTION

Metabolic regulation must coordinate energy production, macromolecule synthesis, and redox balance across multiple tissues and temporal scales to maintain organismal homeostasis (*1*). This coordination becomes particularly critical during physiological challenges, including feeding, fasting, exercise, or disease, when tissues must rapidly adjust their metabolic priorities while maintaining overall energy balance (*1*, *2*). However, understanding how this intertissue metabolic coordination occurs requires measuring actual metabolic pathway activity rather than simply measuring metabolite levels as the same metabolite pools can reflect vastly different flux states depending on the physiological context (*3*, *4*).

Metabolic flux analysis addresses this limitation by quantifying the rates and directions of biochemical reactions within living systems. Stable isotope tracing has emerged as the gold standard for flux analysis in vivo, where labeled nutrients (e.g., ^13^C and ^15^N) are introduced into an organism and their incorporation into downstream metabolites tracked by mass spectrometry (MS) or nuclear magnetic resonance (*5*–*8*). Two distinct experimental approaches serve different experimental goals: Dynamic-state tracing follows time-dependent incorporation of tracers into metabolites of interest after the start of labeling (*9*), which is useful for evaluating the rates of pathways of interest; steady-state tracing maintains constant tracer levels until equilibrium, enabling estimation of the fractional nutrient contributions to downstream metabolites and pathways comparisons between tissues under physiological conditions (*10*).

Several delivery routes exist for in vivo isotope administration. Tail vein catheterization allows for the continuous infusion of tracers under anesthesia (*5*) and has been used for the study of glucose, lactate, glutamine, and other metabolites in mice (*11*–*13*). While anesthesia may mimic metabolic studies in patients (*5*), its impact on organismal and tumor metabolism may affect results. As an alternative to tracer infusion, dietary labeling is noninvasive but produces variable intake and complex relabeling patterns (*14*). Jugular vein catheterization allows prolonged delivery without anesthesia but requires complex surgery by skilled personnel and necessitates post-op recovery and analgesia (*15*, *16*). The lack of consensus on optimal delivery methods introduces an important source of methodological variability in metabolic flux studies.

Despite the widespread use of anesthesia in in vivo isotope tracing studies, its effects on physiological and tissue-specific metabolism remain insufficiently characterized. Numerous studies have reported both acute and long-term metabolic effects of anesthesia. However, most have been limited to specific organs such as the brain, heart, or blood (*17*–*20*). A physiological understanding of how anesthesia influences whole-body or organ-specific metabolism remains incomplete. In particular, the global metabolic alterations induced by isoflurane anesthesia across different tissues have not been fully investigated.

A method enabling prolonged tracer delivery without prolonged anesthesia or surgical catheterization would enhance precision, reproducibility, and accessibility of stable isotope tracing analysis in vivo. Here, we developed a tail vein catheter infusion system that enables prolonged isotope delivery in awake mice without the need for surgical implantation. We demonstrate robust cystine tracer incorporation into downstream pathways while minimizing procedural and metabolic perturbations, providing a practical platform for in vivo stable isotope tracing under more physiological conditions.

## RESULTS

### Effects of isoflurane anesthesia on serum metabolites

**Fig. 1.**
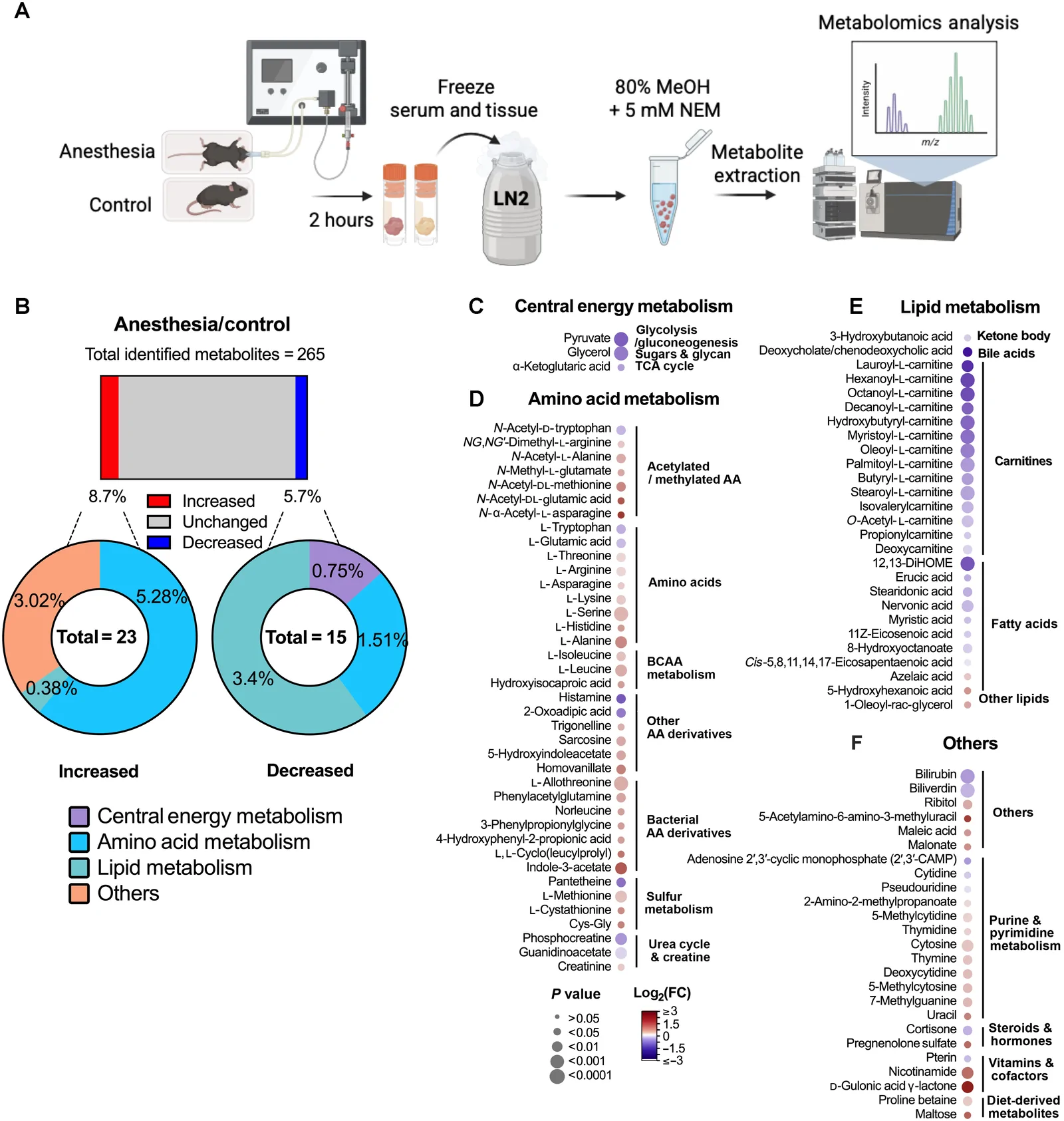
Global serum metabolomic profiling following isoflurane anesthesia. (**A**) Experimental workflow for isoflurane anesthesia and anesthesia-free conditions. Mice were exposed to 2% isoflurane for 2 hours, with body temperature maintained at 37°C using a heating pad and rectal probe monitoring or maintained under anesthesia-free conditions for 2 hours before sample collection. Serum and tissues were immediately snap frozen in liquid nitrogen, extracted with 80% methanol containing 5 mM NEM, and analyzed by liquid chromatography–mass spectrometry (LC-MS)–based metabolomics. Created in BioRender. Y. Kim (2026), https://BioRender.com/lw1k852. (**B**) Summary of significantly altered serum metabolites in the isoflurane anesthesia group relative to anesthesia-free controls (fold change ≥ 2, *P* < 0.05). Bar graphs indicate the numbers of increased and decreased metabolites, and donut charts show their distribution across functional categories (central energy, amino acids, lipid metabolism, and others). (**C** to **F**) Dot heatmaps showing significant serum metabolite changes grouped by functional categories in isoflurane anesthesia compared with anesthesia-free controls. Metabolites with *P* < 0.05 from volcano plot analysis were included. Dot size indicates −log_10_(*P* value), and dot color reflects log_2_(fold change), with red indicating increased abundance and blue indicating decreased abundance under isoflurane anesthesia relative to control conditions (scale, −3.0 to 3.0). Data are from *n* = 10 mice per group (5 males and 5 females). AA, amino acids; FC, fold change.

To test the impact of isoflurane anesthesia on metabolism, mice (*n* = 10 per group, 5 males and 5 females) were subjected to isoflurane anesthesia for 2 hours on a heating pad or control conditions, followed by serum and organ collection. Metabolites were extracted using 80% methanol containing 5 mM *N*-ethylmaleimide (NEM), which covalently reacts with free thiols such as cysteine and glutathione, thereby preventing artifactual oxidation and allowing accurate measurement of sulfur metabolites, followed by analysis by liquid chromatography–high-resolution mass spectrometry (LC-HRMS) (Fig. 1A).

Global metabolomics analysis revealed extensive alterations between the isoflurane anesthesia group and the anesthesia-free control group. Among 265 serum metabolites identified, 38 metabolites showed at least a twofold change under isoflurane anesthesia (*P* < 0.05). Of these, 23 metabolites increased, and 15 metabolites decreased (Fig. 1B). Among the 23 increased metabolites, amino acid metabolites represented the largest proportion (5.28%), followed by other metabolites (3.02%) and lipid metabolites (0.38%). Among the 15 decreased metabolites, lipid metabolites represented the largest proportion (3.4%), followed by amino acid metabolites (1.51%) and central energy metabolites (0.75%) (Fig. 1B).

Dot heatmaps revealed coordinated metabolic alterations across central energy, amino acid, lipid, and other pathways in response to isoflurane anesthesia (Fig. 1, C to F). In the central energy category (Fig. 1C), pyruvate, glycerol, and tricarboxylic acid (TCA) cycle intermediates including α-ketoglutaric acid were decreased under isoflurane anesthesia. Moreover, amino acid metabolism showed widespread alterations under isoflurane anesthesia (Fig. 1D). Most of proteinogenic amino acids were increased, together with several acetylated and methylated amino acid derivatives. Branched-chain amino acid (BCAA)–associated metabolites, including leucine, l-isoleucine, and hydroxyisocaproic acid, were among the increased metabolites. Sulfur-related metabolites including Cys-Gly, l-cystathionine, and l-methionine were likewise elevated. Additional amino acid derivatives were also altered. Together, these findings indicate broad alterations in amino acid-associated metabolites under isoflurane anesthesia.

Lipid metabolism also showed notable alterations under isoflurane anesthesia (Fig. 1E). Acylcarnitine species across multiple chain lengths were consistently decreased, including hexanoyl-l-carnitine, lauroyl-l-carnitine, palmitoyl-l-carnitine, and oleoyl-l-carnitine. Fatty acid–associated metabolites were also generally decreased, although the pattern was less uniform than that observed for acylcarnitines. Bile acids including deoxycholate and chenodeoxycholic acid were likewise decreased. Together, these findings indicate substantial remodeling of lipid-associated metabolites under isoflurane anesthesia, with the most consistent changes observed in acylcarnitine species.

In the “others” category (Fig. 1F), purine and pyrimidine metabolites showed mixed directionality, with increases in uracil, 7-methylguanine, 5-methylcytosine, deoxycytidine, thymine, cytosine, thymidine, and 5-methylcytidine, whereas cytidine, pseudouridine, and adenosine 2′,3′-cyclic monophosphate were decreased. The broad increase in methylated nucleobases and nucleosides, including 5-methylcytosine, 5-methylcytidine, and 7-methylguanine, alongside decreased cytidine and pseudouridine, may reflect altered nucleotide salvage activity or changes in RNA turnover under anesthesia, although further investigation would be required to distinguish these possibilities. Steroid and hormone-related metabolites were also altered, with increased pregnenolone sulfate and decreased cortisone levels, suggesting complex remodeling of steroid metabolism under anesthesia. To determine whether prolonged isoflurane exposure altered systemic stress responses, we assessed corticosterone levels in this serum dataset (fig. S1). No significant differences were observed between isoflurane anesthesia and control groups when all animals were analyzed together (fig. S1A). However, sex-stratified analysis revealed higher baseline corticosterone levels in female mice and opposite responses to isoflurane between males and females, with decreased corticosterone levels in males and increased levels in females (fig. S1B). Together, these findings reveal sex-dependent modulation of steroid hormone responses to isoflurane anesthesia, highlighting the importance of considering biological sex as a variable in the interpretation of anesthesia-induced metabolic changes.

### Organ-specific metabolic alterations are induced by isoflurane anesthesia

**Fig. 2.**
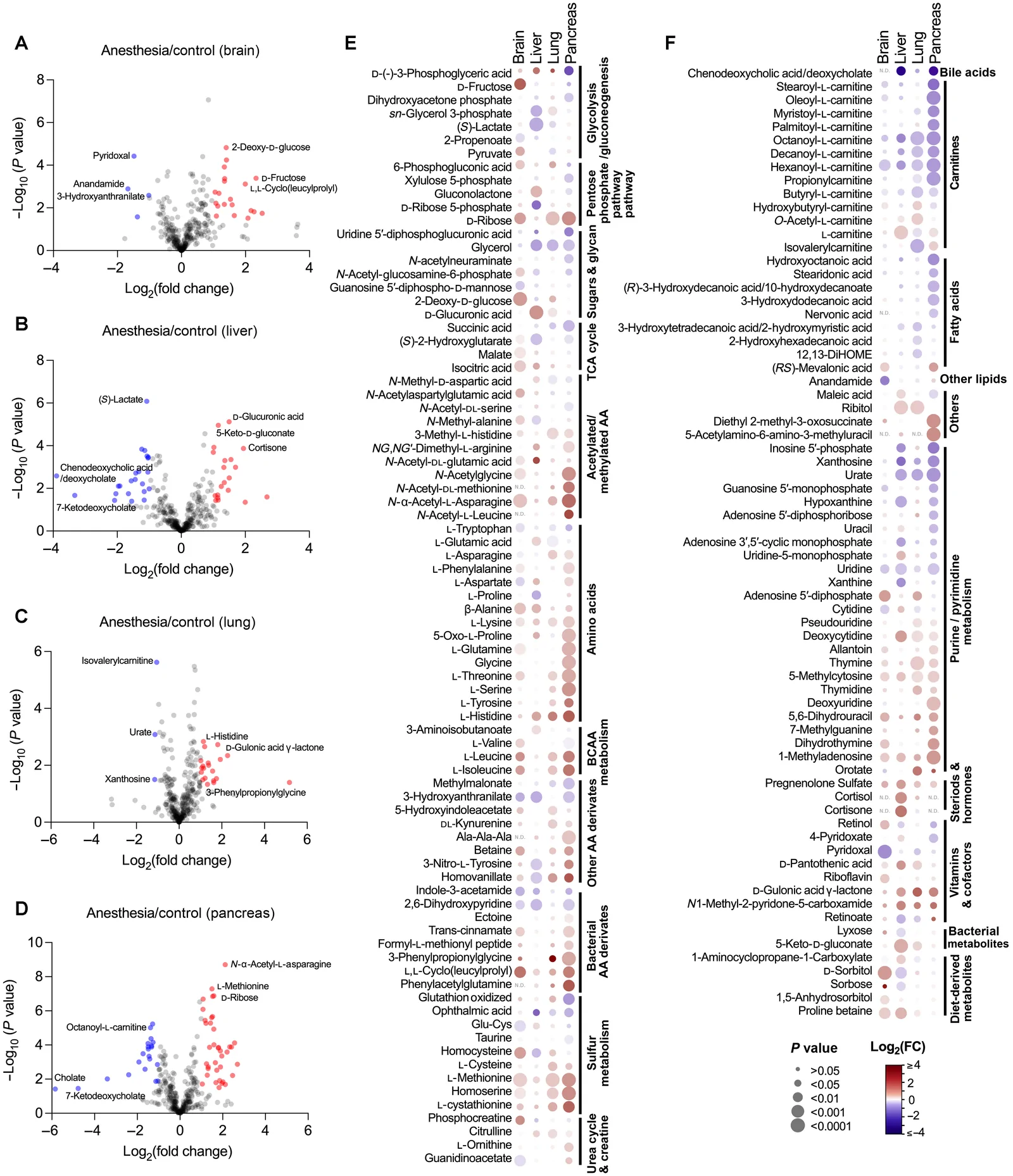
Metabolomic changes across brain, lung, liver, and pancreas induced by anesthesia. Metabolite alterations in the brain, lung, liver, and pancreas following 2 hours of isoflurane anesthesia relative to anesthesia-free controls. Metabolites were profiled by LC-MS. (**A** to **D**) Volcano plots of global metabolomics in the (A) brain, (B) lung, (C) liver, and (D) pancreas. Metabolites with significantly increased abundance are shown in red and decreased metabolites in blue, with representative metabolites labeled. Plots display log_2_ (fold change) versus −log_10_ (*P* value) for isoflurane anesthesia compared to anesthesia-free controls. (**E** and **F**) Dot heatmaps showing metabolic changes grouped by functional categories across the brain, lung, liver, and pancreas. Metabolites were included if they reached *P* < 0.01 in at least one of the examined tissues. Dot size indicates −log_10_(*P* value), and dot color represents log_2_(fold change) in isoflurane anesthesia relative to control conditions (scale, −4 to 4). Data are from *n* = 10 mice per group (5 males and 5 females). AA, amino acids; FC, fold change.

The changes in serum metabolites may reflect altered metabolism in metabolically active organs or disrupted substrate delivery to these tissues. To investigate organ-level metabolic responses, we conducted metabolite profiling across four organs, which revealed both shared and tissue-specific metabolic alterations induced by isoflurane anesthesia compared to anesthesia-free controls (Fig. 2, A to F).

The brain exhibited a marked metabolic response to anesthesia, characterized by broad increases in intermediates of glycolysis and gluconeogenesis, including pyruvate and d-fructose (Fig. 2, A and E). Concurrent increases were observed across the pentose phosphate pathway, including d-ribose and 6-phosphogluconate, as well as multiple sugar and glycan metabolites. Several amino acids and amino acid derivatives were also increased in the brain. These increases occurred alongside decreased acylcarnitines across multiple chain lengths, indicating suppression of mitochondrial fatty acid oxidation. Additionally, the endocannabinoid anandamide was significantly decreased in brain, alongside reductions in pyridoxal, pointing to anesthesia-induced perturbations in neurochemical signaling beyond core energy metabolism. Together, the brain metabolic profile suggests a fundamental reorientation of cerebral energy metabolism away from fatty acid oxidation toward glycolytic and biosynthetic pathways under isoflurane anesthesia.

The liver showed a distinct metabolic response to anesthesia (Fig. 2B). The most significant increases were observed in d-glucuronic acid and 5-keto-d-gluconate, consistent with active glucuronidation and hepatic detoxification, likely reflecting that the liver is a primary site of isoflurane metabolism, whereas lactate was decreased. TCA cycle intermediates showed mixed changes of generally small magnitude, suggesting subtle remodeling of oxidative metabolism. Bile acids including chenodeoxycholic acid/deoxycholate and 7-ketodeoxycholate were significantly decreased, pointing to disrupted hepatic metabolism. Cortisone, cortisol, and pregnenolone sulfate were increased. Notably, amino acid metabolism, acylcarnitines, and fatty acids were largely unchanged in the liver, in marked contrast to other tissues, suggesting that many hepatic metabolic processes are largely maintained under isoflurane anesthesia.

Despite being the primary route of isoflurane delivery and, therefore, exposed to the highest local drug concentrations, the lung showed the least extensive metabolic response of all tissues examined (Fig. 2C). Only a small number of metabolites reached statistical significance, including increases in l-histidine, d-gulonic acid γ-lactone, and 3-phenylpropionylglycine and decreases in isovalerylcarnitine, urate, and xanthosine. Although unexpected, this may reflect the fact that isoflurane undergoes minimal pulmonary metabolism.

The most extensive and distinctive tissue-specific response was observed in the pancreas (Fig. 2, D to F). First, free amino acids were broadly and significantly increased, spanning nearly all proteinogenic amino acids including glycine, serine, threonine, glutamine, histidine, tyrosine, phenylalanine, asparagine, tryptophan, proline, lysine, arginine, and glutamic acid, as well as BCAAs leucine and isoleucine. Notably, sulfur amino acid metabolites were also increased, including methionine, cysteine, and cystathionine. This amino acid accumulation was accompanied by broad increases in acetylated and methylated amino acid derivatives, including *N*-alpha-acetyl-l-asparagine among others, suggesting widespread perturbation of amino acid metabolism and processing. Second, acylcarnitines were broadly decreased across multiple chain lengths, consistent with the cross-tissue pattern of suppressed mitochondrial fatty acid oxidation. Third, purine and pyrimidine metabolites were most profoundly altered in the pancreas compared to other tissues, with broad suppression of purine nucleotides and catabolic intermediates shared with the liver, accompanied by a uniquely pancreas-specific divergent increase in specific pyrimidine species including orotate, dihydrouracil, thymidine, and deoxyuridine. Bile acids including cholate and 7-ketodeoxycholate were also decreased. Together, these findings indicate that isoflurane anesthesia induces coordinated metabolic alterations across multiple organs while maintaining distinct tissue-specific metabolic signatures.

### Tail vein infusion provides physiological tracer delivery

Stable isotope infusion in mice has traditionally been performed either through tail vein infusion under anesthesia or through surgically implanted jugular catheters. Both approaches introduce metabolic disturbances, either through anesthetic exposure or through surgical stress and postoperative recovery, and they also present technical challenges that limit reproducibility and their broad applicability across research labs. To overcome these limitations, we developed a tail vein catheter infusion method that enables stable isotope delivery on conscious mice without the need for surgical implantation.

**Fig. 3.**
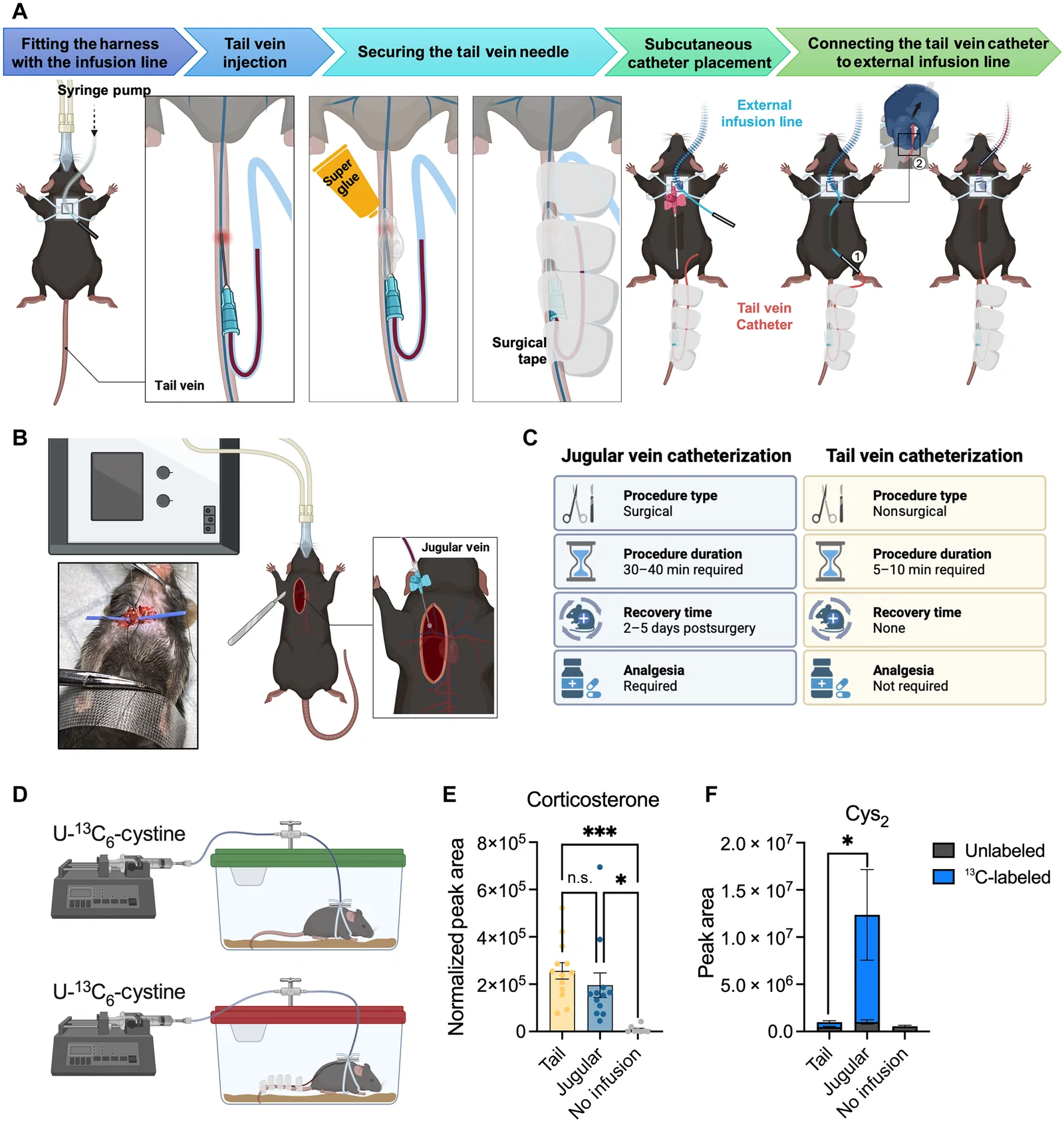
Comparison of jugular and tail vein catheterization for U-^13^C_6_-cystine infusion. (**A**) Workflow of the tail vein catheter infusion setup. The external infusion line is threaded through a mouse harness and connected to the lateral tail vein catheter, stabilized with cyanoacrylate adhesive and surgical tape. A subcutaneous tunnel is created from the dorsal skin to the tail base to guide and secure the infusion line. (1) The external infusion through the tunnel and connected to the catheter via a blunted connector. (2) The line is gently pulled to position the junction and complete the infusion circuit. (**B**) Schematic of surgically implanted jugular vein catheterization requiring prolonged anesthesia. (**C**) Comparison of jugular and tail vein catheterization characteristics, including procedure type, setup duration, recovery requirement, and analgesia requirement. (**D**) Experimental setup for tracer delivery. Following catheter placement and recovery, U-^13^C_6_-cystine (10 mg/ml) was infused at 120 μl/hour for 4 hours. At the end of infusion, serum and tissues were collected, snap frozen in liquid nitrogen, and processed for metabolomics. Serum was extracted with 80% methanol containing NEM to stabilize sulfur metabolites and analyzed by LC-MS in positive ion mode. (**E**) Serum corticosterone levels following infusion. Statistical significance was determined using one-way analysis of variance (ANOVA) followed by Tukey’s multiple comparisons test. ***P < 0.001 for no infusion versus tail infusion comparisons; *P < 0.05 for no infusion versus jugular infusion comparisons; n.s., not significant for tail versus jugular comparisons. (**F**) Absolute cystine peak area in serum showing unlabeled and ^13^C-labeled cystine across groups. Data are means ± SEM (*n* = 12 for jugular, *n* = 13 for tail, and *n* = 8 for no infusion) with each dot representing an individual mouse. Statistical significance was determined using a two-tailed unpaired Student’s *t* test. *P < 0.05 for tail versus jugular comparisons. Created in BioRender. Y. Kim (2026), https://BioRender.com/lw1k852.

We established a standardized tail vein catheterization workflow for awake infusion studies. The procedure began by fitting mice with a harness through which the external infusion line from the pump was threaded. A 27-gauge catheter needle was inserted into the lateral tail vein, secured with a small drop of cyanoacrylate adhesive, and reinforced with surgical tape to stabilize the intravenous access point (Fig. 3A). A temporary subcutaneous tunnel was created by inserting a 16-gauge introducer catheter through the dorsal skin and advancing it subcutaneously toward the base of the tail. This tunnel allowed the external infusion line to be guided from the dorsal entry point through the subcutaneous tract and out near the tail base. Once the infusion line emerged at the tail base, the introducer was removed, and the infusion line was connected to the tail vein catheter via a blunted connector, as shown in Fig. 3A. Following catheter setup, mice were returned to infusion cages and allowed to recover from brief isoflurane exposure before connection to the infusion pump and initiation of tracer delivery.

To determine whether the brief isoflurane exposure required for tail vein catheter setup induces persistent metabolic perturbations, mice were assigned to handled control, brief isoflurane (10 min), or catheter implantation (under brief isoflurane exposure) groups. Following each treatment condition, mice were returned to a clean cage for 1 hour before serum collection and metabolomic analysis, together with serum samples from the prolonged isoflurane exposure group (2 hours). Principal components analysis (PCA) demonstrated that the brief isoflurane and catheter implantation groups clustered closely with handled controls, whereas prolonged isoflurane exposure formed a distinct cluster (fig. S2A). Hierarchical clustering analysis showed a similar pattern (fig. S2B). These findings indicate that the extensive metabolic remodeling observed with prolonged isoflurane exposure is not recapitulated following brief setup-related procedures. In corticosterone analysis, overall corticosterone levels in the brief isoflurane and catheter implantation groups remained comparable to handled controls (fig. S2C).

We next compared jugular vein and tail vein catheterization as delivery routes for U-^13^C_6_-cystine infusion, which we previously established for in vivo isotope tracing (*21*). Jugular catheterization requires 30 to 40 min of operative time for surgical implantation of a catheter through the right cervical region (Fig. 3B), prolonged isoflurane exposure, 2 to 5 days of postoperative recovery, and analgesic administration (Fig. 3C). In contrast, tail vein catheterization represented a nonsurgical approach completed within 5 to 10 min under brief isoflurane exposure and does not require postoperative recovery or analgesic treatment (Fig. 3C). Following recovery from surgery or brief anesthesia, jugular- and tail vein–catheterized mice were individually placed into infusion cages and connected to a programmable pump system delivering isotope tracer at a constant infusion rate for the designated infusion period (Fig. 3D). Tail vein–catheterized mice exhibited spontaneous movement, exploratory activity, and grooming behaviors throughout the infusion period (movies S2 to S4). Similar behaviors were observed in age-matched control mice maintained under standard home-cage conditions (movie S1).

Serum corticosterone levels were significantly elevated in both tail vein and jugular infusion groups relative to no-infusion controls but did not differ between infusion routes (Fig. 3E), suggesting comparable physiological stress responses between the two infusion approaches. Measurement of circulating cystine pools showed that jugular infusion significantly increased both labeled and unlabeled cystine pools, resulting in a marked increase of the total circulating cystine pool compared with tail infusion or no-infusion controls. In contrast, tail vein infusion maintained circulating cystine levels closer to physiological baseline conditions (Fig. 3F).

Together, these findings indicate that tail vein catheterization provides a minimally invasive and physiologically faithful platform for stable isotope infusion. Compared with jugular catheterization, this approach reduces procedural burden, eliminates surgical recovery, and preserves endogenous metabolite pool sizes while supporting robust isotopologue analysis.

### Tail vein infusion provides stable labeling of cystine-derived metabolites in tissues

**Fig. 4.**
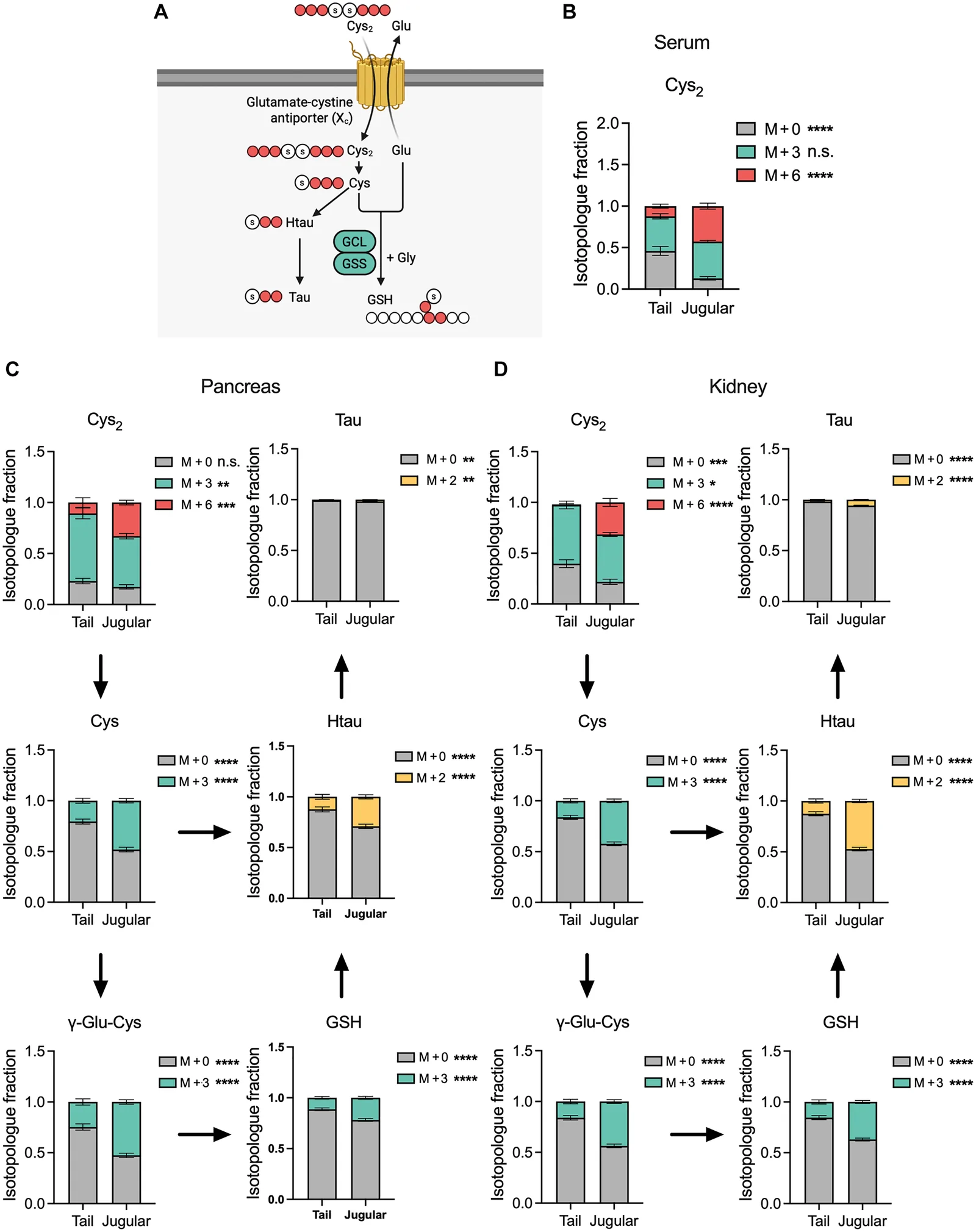
Isotopologue tracing of cystine incorporation into glutathione and taurine pathways. (**A**) Schematic of cystine utilization pathway, illustrating incorporation into cysteine, γ-glutamylcysteine, glutathione, hypotaurine, and taurine. Labeled carbon atoms from U-^13^C_6_-cystine are indicated in red. Created in BioRender. Y. Kim (2026), https://BioRender.com/lw1k852. (**B** to **D**) Isotopologue analysis of serum, pancreas, and kidney collected after U-^13^C_6_-cystine infusion via jugular or tail vein catheterization. (B) Serum cystine isotopologue distribution (M + 0, M + 3, and M + 6). (C and D) Isotopologue fractions of cystine-derived metabolites in pancreas (C) and kidney (D), showing incorporation into cysteine, γ-glutamylcysteine, glutathione, hypotaurine, and taurine. Data are presented as means ± SEM (*n* = 12 for jugular and *n* = 9 for tail). Statistical significance was determined using a two-tailed unpaired Student’s *t* test. *P < 0.05; **P < 0.01; ***P < 0.001; ****P < 0.0001; n.s., not significant.

Incorporation of U-^13^C_6_-cystine into downstream metabolites, including cysteine, γ-glutamylcysteine, glutathione, hypotaurine, and taurine, was examined (Fig. 4A). Red carbon atoms indicate the expected ^13^C labeling patterns, with U-^13^C_6_-cystine generating distinct isotopologue species (M + 2, M + 3, and M + 6) that enable quantitative tracing of cystine utilization.

To evaluate tracer labeling achieved by tail vein versus jugular vein catheterization, serum and tissues were collected immediately after the infusion period, snap frozen in liquid nitrogen, stored at −80°C, and later extracted with 80% methanol containing 5 mM NEM before liquid chromatography–mass spectrometry (LC-MS) analysis. In serum, isotopologue analysis of circulating cystine showed a significantly reduced M + 0 fraction and significantly increased M + 6 fraction during jugular infusion compared with tail vein infusion (Fig. 4B), consistent with differences in tracer pool size. Tail infusion maintained a substantial unlabeled fraction, with total ^13^C labeling (M + 3 and M + 6) of roughly 44%, whereas jugular infusion drove cystine labeling to nearly 87%. Thus, tail vein catheterization achieved sufficient tracer incorporation for downstream isotopologue analysis.

In pancreas, tissue cystine (Cys2) was highly labeled under both infusion conditions, with total ^13^C-labeled fractions remaining high in both tail and jugular infusion groups. However, jugular infusion showed a greater M + 6 fraction compared with tail vein infusion (Fig. 4C), consistent with serum labeling patterns (Fig. 4B). In kidney, jugular infusion resulted in higher total ^13^C labeling together with greater M + 6 enrichment compared with tail vein infusion (Fig. 4D). These findings indicate substantial tracer incorporation into tissue cystine pools under both infusion conditions while also revealing route-dependent differences in isotopologue distribution and labeling extent. For downstream metabolites, including cysteine, γ-glutamylcysteine, glutathione, hypotaurine, and taurine, overall isotopologue propagation patterns were similar between infusion routes, although jugular infusion consistently produced greater downstream isotopologue enrichment than tail vein infusion. To further evaluate whether the marked increase in circulating cystine observed with jugular infusion was reflected in tissues, downstream metabolite isotopologue fractions were normalized to tissue cystine enrichment, and total metabolite pool sizes (^12^C and ^13^C pools) were quantified for cystine-derived metabolites in pancreas and kidney (fig. S3). Following normalization, several downstream metabolites continued to exhibit significantly greater isotopologue enrichment in the jugular group compared with the tail vein group (fig. S3A), suggesting that differences in tracer delivery and local precursor availability may continue to influence downstream tracer incorporation. In both pancreas and kidney, total cystine pools were higher in the jugular group than in the tail vein group and were accompanied by increased M + 6 cystine pools (fig. S3B). Downstream sulfur metabolites exhibited metabolite- and tissue-specific changes in total pool size, but labeled metabolite pools were generally increased in the jugular group. In pancreas, total cysteine and γ-glutamylcysteine pools were increased in the jugular group together with corresponding increases in M + 3 pools. In contrast, total glutathione and hypotaurine pools were reduced in the jugular group despite significantly increased ^13^C-labeled fractions, whereas taurine pools remained largely similar between groups with increased M + 2 taurine pools in the jugular group. In kidney, total cysteine and γ-glutamylcysteine pools were not substantially different between groups despite increased M + 3 pools, consistent with corresponding reductions in endogenous M + 0 pools. Glutathione, hypotaurine, and taurine showed marked increases in ^13^C-labeled pools in the jugular group, with hypotaurine exhibiting the strongest increase accompanied by expansion of both M + 0 and M + 2 pools.

To determine whether prolonged isoflurane exposure directly alters tracer behavior during infusion studies, we compared awake mice (awake group) and mice maintained under isoflurane-anesthetized infusion conditions (asleep group) receiving identical tail vein U-^13^C_6_-cystine infusions for 4 hours while maintaining body temperature at 37°C throughout anesthesia. Overall isotopologue distributions of serum cystine and tissue cystine-derived metabolites were largely comparable between conditions, with most isotopologue fractions showing no significant differences between the awake and asleep groups (fig. S4, A and B). In contrast, metabolite pool-size analysis revealed selective tissue- and metabolite-specific differences between the awake and asleep groups (fig. S4, C and D). The serum cystine total pool size was increased in the asleep group and was accompanied by increased M + 3 cystine pools, whereas increases in M + 6 cystine pools did not reach statistical significance. In pancreas, most cystine-derived metabolite pools were not substantially altered between the awake and asleep groups, although hypotaurine pools were reduced in the asleep group. In kidney, labeled cysteine and glutathione pools were increased in the asleep group, whereas hypotaurine and taurine exhibited increases in both labeled and total metabolite pools.

Together, these findings indicate that tail vein infusion supports robust and informative labeling of cysteine and downstream sulfur metabolites while maintaining circulating cystine pools closer to physiological levels, whereas prolonged isoflurane exposure primarily influences metabolite abundance rather than overall cystine tracer incorporation patterns. In contrast, jugular infusion produced greater isotopologue enrichment together with substantial tracer pool expansion and required surgical catheterization. Thus, tail vein infusion provides a technically simpler and more physiologically faithful approach that still enables informative isotopologue-based pathway analysis.

## DISCUSSION

As in vivo metabolomics and stable isotope tracing continue to expand, achieving physiologically accurate measurements in mouse models has become increasingly important (*8*, *22*). These experimental approaches have traditionally relied on dietary labeling (*14*, *23*), tail vein infusions performed under continuous anesthesia (*5*), or surgical jugular catheterization for prolonged tracer delivery (*24*–*26*). However, each of these approaches introduces physiological or technical variables that complicate accurate interpretation of in vivo flux. Dietary labeling results in uncontrolled nutrient intake and complex relabeling patterns. Tail vein delivery has also been used for steady-state isotope infusion, but these procedures are performed under continuous anesthesia (*5*), which introduces physiological disturbances and alters systemic metabolism. Jugular catheter infusion can achieve steady state labeling but necessitates surgery, prolonged anesthesia, external thermoregulation to prevent hypothermia, postoperative recovery, and analgesic administration, all of which introduce physiological perturbations (*15*, *16*). Despite the widespread use of anesthesia in these procedures, its organ-level metabolic effects were incompletely characterized. Here, we systematically defined how isoflurane anesthesia reprograms systemic metabolism and established a nonsurgical tail vein catheter infusion platform that enables physiological steady state isotope tracing on conscious animals while minimizing procedural and metabolic disruption.

The most consistent cross-tissue impact of anesthesia was broad suppression of acylcarnitines and nucleotides and accumulation of amino acids. This metabolic rewiring aligns with known mechanisms of isoflurane action and with previous reports documenting anesthesia associated metabolic perturbations. Isoflurane inhibits mitochondrial complex I, decreasing oxygen consumption and adenosine 5′-triphosphate production (*27*, *28*), and prior studies have linked isoflurane and surgical stress to disruption of fatty acid and carbon metabolism (*18*). BCAA oxidation is rapid and dynamically regulated across tissues (*29*), and oxidative BCAA disposal is influenced by the overall oxidative activity of mitochondria within each tissue (*30*). Thus, although BCAA oxidation was not directly measured in our study, the observed BCAA elevations raise the possibility that isoflurane-induced mitochondrial inhibition may contribute to altered BCAA catabolism. Acute alterations in serum metabolites observed here, including changes in lactate and fatty acid–associated metabolites, are consistent with findings showing that diverse anesthetics and sampling procedures immediately distort circulating metabolites (*31*). Additional studies comparing awake versus isoflurane anesthesia conditions in the prefrontal cortex similarly reported increased nucleosides under anesthesia (*19*), reinforcing that anesthesia represents a metabolic state distinct from wakefulness. Furthermore, prior work using isoflurane and surgical stress reported acute and long-lasting transcriptomic remodeling in the aged heart, including maladaptive metabolic reprogramming (*18*). Together with our findings, these studies further support that anesthesia is associated with broad metabolic alterations across multiple biological contexts. While we did not directly measure circulating insulin, glucagon, or catecholamines, the broad amino acid and lipid changes observed across tissues may also reflect not only direct mitochondrial effects of isoflurane but also indirect consequences mediated through hormonal and autonomic pathways that are disrupted under anesthesia.

Beyond this shared response, each tissue exhibited a distinct metabolic signature. The brain showed glycolytic and pentose phosphate pathway metabolite accumulation alongside suppressed acylcarnitines, consistent with compensatory up-regulation of nonoxidative energy metabolism in response to mitochondrial inhibition and aligning with prior reports of altered cerebral metabolism under isoflurane (*17*, *19*). The liver alterations involved glucuronidation and detoxification, consistent with its role as the primary site of isoflurane metabolism via cytochrome P450 enzymes (*32*), accompanied by disruption of bile acid metabolism that may reflect altered hepatic metabolism. Paradoxically, the lung showed the least metabolic perturbation of all tissues despite direct anesthetic exposure, suggesting that local exposure alone was not sufficient to drive broad metabolic remodeling in this tissue.

The most notable tissue-specific finding was a near-global accumulation of free amino acids in the pancreas to a greater extent than any other tissue. Notably, serum also showed broad increases in amino acids and BCAAs under isoflurane anesthesia, suggesting that the pancreatic accumulation may, in part, reflect a systemic shift in amino acid metabolism. Several mechanisms could contribute to this systemic pattern. Altered mitochondrial oxidative metabolism may contribute to BCAA accumulation by affecting oxidative BCAA disposal (*30*), although this mechanism remains to be directly tested. Broader amino acid accumulation may also reflect systemic changes in amino acid utilization under anesthesia. The particularly pronounced accumulation in the pancreas may additionally reflect local factors including vagal suppression reducing exocrine protease secretion or impaired endocrine-driven amino acid clearance. Whether the pancreatic pattern represents an amplification of a systemic response or a superimposed local mechanism warrants further investigation.

To address these metabolic perturbations, we developed a nonsurgical tail vein catheter infusion system requiring only 5 to 10 min of isoflurane exposure, after which mice recover rapidly and remain freely moving throughout infusion. Metabolomic profiling confirmed that brief isoflurane exposure and catheter setup produced profiles clustering closely with handled controls. While prior reports suggest that isoflurane can acutely alter whole-body leucine, glucose, and fatty acid metabolism (*33*), our findings indicate that these effects are rapidly reversible following brief isoflurane exposure. A recently published protocol has similarly described awake tail vein infusion (*34*), and our approach extends this method with subcutaneous line routing and harness-tether integration to minimize disruption during prolonged infusion.

Comparison of jugular and tail vein infusion revealed that jugular infusion produced an approximately eightfold expansion of circulating cystine pools, whereas tail infusion maintained levels closer to physiological baseline. This likely reflects differences in delivery kinetics between routes and potentially saturation of the xCT cystine transporter (SLC7A11) at higher circulating concentrations achieved by jugular infusion. Consistent with this, despite large differences in circulating cystine, tissue cystine abundance was substantially less divergent between routes, suggesting partial buffering at the tissue level. Despite these differences in pool size and labeling extent, isotopologue propagation patterns into downstream sulfur metabolites were broadly similar between routes, indicating that tail vein infusion supports pathway analysis while preserving more physiological metabolite pool sizes.

Direct comparison of awake and anesthetized tail vein infusion revealed that isotopologue distributions were broadly comparable between conditions, whereas selective pool size differences were observed under anesthesia. This dissociation suggests that isoflurane primarily alters metabolite abundance rather than fundamentally disrupting tracer incorporation. However, these conclusions are based solely on cystine as a tracer and may not generalize to other metabolic contexts. Given that isoflurane alters nucleotide pools, TCA cycle intermediates, and amino acid abundance, tracers interrogating these pathways, such as those used to assess nucleotide salvage versus de novo synthesis (*35*), or carbon source contributions to the TCA cycle (*36*), may be more substantially affected by anesthesia-induced changes in substrate availability and pool sizes. Several additional experimental variables warrant consideration, including duration of food withdrawal, stress induced by the procedure, infusion duration, choice of anesthetic agent (*17*), and analgesic administration in surgical models, whose metabolic consequences remain incompletely characterized.

In summary, this study provides a multiorgan characterization of isoflurane-induced metabolic remodeling and demonstrates that nonsurgical tail vein catheter infusion reduces anesthesia- and surgery-associated perturbations while supporting robust stable isotope tracing in conscious mice. The organ-specific metabolic signatures identified here, particularly the notable amino acid accumulation in the pancreas and glycolytic shift in the brain, highlight the importance of tissue-level profiling in understanding the systemic consequences of anesthetic exposure.

## MATERIALS AND METHODS

### Animal care

All animal experiments were conducted in accordance with the ethical guidelines and approval of the University of South Florida Institutional Animal Care and Use Committee (protocols IS00012540R and IS00008736R). C57BL/6NJ mice (Jackson Laboratory, strain no. 005304) were housed in the Animal Research Facility at the Moffitt Cancer Center under specific pathogen–free conditions with a 12-hours light/dark cycle (lights on at 6:00 a.m. and off at 6:00 p.m.), ambient temperature of 21° to 23°C, and relative humidity maintained at 30 to 70%. Mice had ad libitum access to standard chow (Envigo Bioproducts Inc., T.2918.CS) and water.

### Mice

C57BL/6NJ mice at 12 weeks of age were used for all experiments unless otherwise noted, with the exception of jugular versus tail vein infusion comparison studies, which were performed in 16- to 17-week-old mice. Both sexes were included in equal proportions across experimental groups. Mice were randomly assigned across experimental groups. In all experiments, mice were not prefasted, and both food and water access were withheld uniformly across all groups during the experimental procedures. At the end of each experimental procedure, blood was collected via the submandibular vein into serum separator tubes (BD, catalog no. 365967) and centrifuged at 10,000 rpm for 15 min at room temperature. The serum fraction was transferred to a 1.5-ml tube, snap frozen in liquid nitrogen, and stored at −80°C until metabolite extraction. Immediately following blood collection, mice were euthanized by cervical dislocation. Tissues were rapidly excised, placed in prelabeled cryovials, snap frozen in liquid nitrogen, and stored at −80°C until analysis.

### Anesthesia

For the anesthesia condition, mice were exposed to 2% isoflurane delivered through a nose cone for 2 hours. Throughout the procedure, mice were placed on a heating pad maintained at 37°C to prevent hypothermia. Body temperature was monitored rectally every 20 min using a calibrated probe thermometer to ensure that normothermia was maintained throughout the anesthesia period. The anesthesia-free control mice were transferred from their home cage to an identical experimental cage without access to food or water for the same 2-hour period. All mice were continuously monitored to ensure normal respiration and to minimize stress. At the end of the 2-hour period, blood and tissues were rapidly collected, placed in prelabeled cryovials, and flash frozen in liquid nitrogen for subsequent LC-MS analysis.

### Stable isotope animal infusions

To prepare the ^13^C_6_-cystine tracer, ^13^C_3_-cysteine (10 mg/ml in sterile saline; Cambridge Isotope Laboratories, CLM-4320-H-PK) was oxidized with an equimolar quantity of H_2_O_2_ for 30 min at room temperature, and the reaction was terminated by heating at 60°C for 5 min. The resulting cystine was resolubilized with 6 mol/liter HCl before infusion. Neutralization to physiological pH was not performed in order to maintain cystine solubility. Jugular vein catheterization and stable isotope infusions were performed as previously described (*21*). Briefly, indwelling catheters were surgically implanted into the right jugular vein 2 to 7 days before infusion, and mice were allowed to recover under standard housing conditions. On the day of infusion, catheters were connected to a syringe pump through a swivel and tether system (SAI Infusion Technologies) that allowed free movement within the cage. A spring-supported harness was used to secure the tether and prevent disconnection.

For tail vein catheter infusions, the infusion line was assembled using a 3-ml syringe (Monoject SoftPack Syringe, Covidien 1180300777) fitted with a 25-gauge needle (Exchange Supplies, code: TO25) connected to precut polyethylene tubing and joined with blunted 25-gauge connectors to ensure continuous flow. The line was filled with tracer solution and checked for patency before use. The completed line was routed through a stainless-steel spring tether. Mice were fitted with a harness, anesthetized with 2% isoflurane, and placed on a heating pad to facilitate vasodilation. A 27-gauge tail vein catheter with butterfly tabs (SAI Infusion Technologies, BF-27-01) removed was inserted into the lateral tail vein and flushed with 0.3 to 0.4 ml of sterile saline to confirm placement. The catheter was fixed with tissue adhesive and secured with surgical tape along the length of the tail. To externalize the infusion line, a 16-gauge Surflash polyurethane I.V. catheter (Terumo, SC-360106) was used to tunnel the tubing subcutaneously from the base of the tail to the mid-back, exiting beneath the harness connection point. The tail catheter was then attached to the infusion line via a blunted 25-gauge connector, drawn into a protective metal spring tether, and connected to a programmable syringe pump (BS-8000, Braintree Scientific). As with jugular catheter infusions, the tubing and pump were integrated into a swivel and spring-supported tether system (SAI Infusion Technologies) that allowed mice to move freely within the cage throughout the infusion period.

To evaluate the effect of isoflurane anesthesia on tracer labeling during tail vein infusion, a separate cohort of mice received U-^13^C_6_-cystine via tail vein catheterization under either awake or isoflurane-anesthetized conditions. Catheter placement was performed as described above using a 27-gauge butterfly needle catheter and connected with infusion line via a blunted 25-gauge connector. In the anesthetized group, 2% isoflurane was maintained continuously throughout the 4-hour infusion period, and mice were placed on a heating pad to maintain body temperature. The awake group recovered from brief anesthesia immediately after catheter placement and moved freely throughout infusion.

All mice that had been set up for infusion received ^13^C_6_-cystine (10 mg/ml) at a constant rate of 120 μl/hour for 4 hours. During the final minutes of infusion, blood was collected from the submandibular vein, and mice were euthanized by cervical dislocation. Tissues of interest were rapidly excised, placed in cryovials, and flash frozen in liquid nitrogen for subsequent LC-MS analysis.

### Brief isoflurane and catheter implantation experiment

To assess whether brief isoflurane exposure or tail vein catheter placement induces lasting metabolic perturbations, mice were assigned to one of four groups: (i) handled controls, in which mice were transferred from their home cage to an experimental cage without food or water; (ii) brief isoflurane exposure, in which mice received 2% isoflurane for 10 min without any additional procedure; (iii) catheter implantation, in which mice underwent complete tail-vein catheter setup under 2% isoflurane anesthesia for ∼10 min; or (iv) prolonged isoflurane exposure, in which mice were maintained under 2% isoflurane anesthesia for 2 hours. Following the handled control, brief isoflurane, and catheter implantation conditions, mice were transferred to experimental cages with food and water withheld for 1 hour before sample collection. Serum samples from the prolonged isoflurane group were collected immediately following the 2-hour anesthesia exposure. Serum was then collected and snap frozen in liquid nitrogen for global metabolomics analysis.

### Sample preparations

Serum and tissue samples were extracted using 80% methanol-based solvent containing 5 mM NEM prepared in 10 mM ammonium formate (pH 7.0). For anesthesia and anesthesia-free samples, internal standards (Cambridge Isotope Laboratories Inc., catalog no. MSK-CAA) were included in the extraction solvent. For stable isotope infusion samples, the extraction solvent was identical except that internal standards were omitted. Frozen serum aliquots (10 μl) were thawed on ice and mixed with 90 μl of ice-cold extraction solvent by pipetting to fully resuspend the sample. The mixtures were then stored at −80°C overnight. Frozen tissues were pulverized under liquid nitrogen using a prechilled BioPulverizer (BioSpec). Pulverized tissue (50 mg per milliliter of extraction solvent) was transferred into precooled tubes, mixed with 80% methanol containing 5 mM NEM in 10 mM ammonium formate (pH 7.0), and stored at −80°C overnight. The following day, all samples were centrifuged at 17,000*g* for 20 min at 4°C, and clarified supernatants were transferred to LC-MS vials and stored at −80°C until analysis. Samples were assigned coded identifiers before extraction and remained coded throughout LC-MS analysis.

### LC-HRMS analysis and data processing

Metabolite analysis was performed using a Vanquish UPLC system (Thermo Fisher Scientific, Waltham, MA) coupled to a Q-Exactive HF Orbitrap mass spectrometer equipped with a heated electrospray ionization source (Thermo Fisher Scientific). Separation was achieved on an Atlantis Premier BEH Z-HILIC VanGuard FIT column (2.1 mm by 150 mm, 2.5 μm; Waters, Milford, MA) maintained at 30°C. The mobile phases consisted of solvent A (10 mM ammonium carbonate and 0.05% ammonium hydroxide in water) and solvent B (100% acetonitrile). The chromatographic gradient was programmed as follows: 0 min, 80% B; 13 min, 20% B; 15 min, 20% B; followed by reequilibration to initial conditions. The flow rate was 150 μl/min, and the injection volume was 5 μl.

The Q-Exactive HF was operated in positive and negative electrospray ionization modes, acquired in separate runs. The MS1 scan range was mass/charge ratio (*m*/*z*) 65 to 975 for negative mode and *m*/*z* 65 to 950 for positive mode. Full MS scans were collected at a resolution of 120,000 (*m*/*z* 200) with an automatic gain control target of 3 × 10^6^.

Raw data files (.raw) were converted to .cdf format using Xcalibur (version 4.0) and further processed using EI-MAVEN (version 0.12.0). Data processing was performed with default parameters except as follows: ionization mode (positive or negative), isotopic tracer (^13^C), and extracted ion chromatogram (EIC) extraction window of ±10 parts per million. Metabolite identification was based on accurate mass and retention time matched to an established in-house library of authentic standards (*37*). Peak intensities were measured as AreaTop (mean of the three highest points of each EIC). Data from isotope labeling experiments were corrected for natural abundance using IsoCor (version 2.2.0). Absolute quantitation of serum and tissues metabolites was performed using internal standard labeled with stable isotopes.

### Statistical analysis

Statistical analyses were performed using GraphPad Prism version 11.0.2 and MetaboAnalyst 6.0. For metabolomics comparisons between two groups, significance was assessed using a two-tailed unpaired Student’s *t* test. Volcano plots display log_2_(fold change) on the *x* axis and −log_10_(*P* value) on the *y* axis; metabolites with *P* < 0.05 and fold change ≥ 2 were considered significant. Serum dot heatmaps included metabolites with *P* < 0.05. For organ-level dot heatmaps, metabolites were included if they reached *P* < 0.01 in at least one of the examined tissues.

Identified metabolites were classified for dot-heatmap visualization using MetaboAnalyst 6.0–guided pathway annotation followed by manual curation. Metabolite names were cross-referenced using *Mus musculus* KEGG and SMPDB pathway libraries, and database-matched annotations were reviewed in the context of mammalian host metabolism, likely biological origin, and the major biochemical contribution of each metabolite. Metabolites were grouped into broad functional categories to support interpretation of anesthesia-associated changes in the mouse metabolome, with selected study-relevant subpathways, including sulfur metabolism and BCAA metabolism, shown separately. Acetylated and methylated amino acid derivatives were grouped separately based on shared chemical modification patterns. Selected metabolites with diet-related, microbiome-associated, or otherwise ambiguous annotations were assigned to broader descriptive categories where appropriate.

PCA and hierarchical clustering heatmaps were generated using MetaboAnalyst 6.0. Metabolite peak intensity tables were uploaded with samples in rows and metabolites in columns. Data were normalized by the sum of metabolite intensities within each sample and autoscaled by mean centering and dividing by the standard deviation of each metabolite; no data transformation was applied. Blank- or QC-based filtering was not applied because blank and QC samples were not included in this analysis. Low-variance and low-abundance filtering were set to 0%, and no metabolites were removed during the filtering step. Metabolites included in PCA and hierarchical clustering heatmaps were selected by one-way analysis of variance (ANOVA) with FDR correction, using an FDR threshold of 0.01. PC1 and PC2 were displayed with 95% confidence regions.

Data are presented as means ± SEM unless otherwise indicated. Comparisons between two groups were assessed using a two-tailed unpaired Student’s *t* test, and comparisons among three or more groups were analyzed using one-way ANOVA followed by Tukey’s multiple comparisons test. Experiments involving two independent variables were analyzed using two-way ANOVA followed by Fisher’s least significant difference multiple comparisons test. For isotope infusion experiments, mice with less than 20% serum tracer labeling were excluded from downstream analysis because this was considered indicative of unsuccessful infusion. Statistical tests and sample sizes for individual experiments are reported in the corresponding figure legends.

## References

[1] R. J. DeBerardinis, C. B. Thompson, Cellular metabolism and disease: What do metabolic outliers teach us? Cell 148, 1132–1144 (2012). 22424225 10.1016/j.cell.2012.02.032 PMC3337773

[2] C. Adrain, Systemic and cellular metabolism: The cause of and remedy for disease? FEBS J. 288, 3624–3627 (2021). 34152675 10.1111/febs.16033

[3] C. M. Metallo, M. G. Vander Heiden, Understanding metabolic regulation and its influence on cell physiology. Mol. Cell 49, 388–398 (2013). 23395269 10.1016/j.molcel.2013.01.018 PMC3569837

[4] C. R. Bartman, B. Faubert, J. D. Rabinowitz, R. J. DeBerardinis, Metabolic pathway analysis using stable isotopes in patients with cancer. Nat. Rev. Cancer 23, 863–878 (2023). 37907620 10.1038/s41568-023-00632-z PMC11161207

[5] B. Faubert, A. Tasdogan, S. J. Morrison, T. P. Mathews, R. J. DeBerardinis, Stable isotope tracing to assess tumor metabolism in vivo. Nat. Protoc. 16, 5123–5145 (2021). 34535790 10.1038/s41596-021-00605-2 PMC9274147

[6] J. A. Reisz, A. D’Alessandro, Measurement of metabolic fluxes using stable isotope tracers in whole animals and human patients. Curr. Opin. Clin. Nutr. Metab. Care 20, 366–374 (2017). 28768294 10.1097/MCO.0000000000000393 PMC5794022

[7] U. Sauer, Metabolic networks in motion: 13C-Based flux analysis. Mol. Syst. Biol. 2, 62 (2006). 17102807 10.1038/msb4100109 PMC1682028

[8] C. Jang, L. Chen, J. D. Rabinowitz, Metabolomics and isotope tracing. Cell 173, 822–837 (2018). 29727671 10.1016/j.cell.2018.03.055 PMC6034115

[9] C. R. Bartman, D. R. Weilandt, Y. Shen, W. D. Lee, Y. Han, T. TeSlaa, C. S. R. Jankowski, L. Samarah, N. R. Park, V. da Silva-Diz, M. Aleksandrova, Y. Gultekin, A. Marishta, L. Wang, L. Yang, A. Roichman, V. Bhatt, T. Lan, Z. Hu, X. Xing, W. Lu, S. Davidson, M. Wuhr, M. G. Vander Heiden, D. Herranz, J. Y. Guo, Y. Kang, J. D. Rabinowitz, Slow TCA flux and ATP production in primary solid tumours but not metastases. Nature 614, 349–357 (2023). 36725930 10.1038/s41586-022-05661-6 PMC10288502

[10] C. R. Bartman, T. TeSlaa, J. D. Rabinowitz, Quantitative flux analysis in mammals. Nat. Metab. 3, 896–908 (2021). 34211182 10.1038/s42255-021-00419-2 PMC9289955

[11] B. Faubert, K. Y. Li, L. Cai, C. T. Hensley, J. Kim, L. G. Zacharias, C. Yang, Q. N. Do, S. Doucette, D. Burguete, H. Li, G. Huet, Q. Yuan, T. Wigal, Y. Butt, M. Ni, J. Torrealba, D. Oliver, R. E. Lenkinski, C. R. Malloy, J. W. Wachsmann, J. D. Young, K. Kernstine, R. J. DeBerardinis, Lactate metabolism in human lung tumors. Cell 171, 358–371.e9 (2017). 28985563 10.1016/j.cell.2017.09.019 PMC5684706

[12] A. Tasdogan, B. Faubert, V. Ramesh, J. M. Ubellacker, B. Shen, A. Solmonson, M. M. Murphy, Z. Gu, W. Gu, M. Martin, S. Y. Kasitinon, T. Vandergriff, T. P. Mathews, Z. Zhao, D. Schadendorf, R. J. DeBerardinis, S. J. Morrison, Metabolic heterogeneity confers differences in melanoma metastatic potential. Nature 577, 115–120 (2020). 31853067 10.1038/s41586-019-1847-2 PMC6930341

[13] C. T. Hensley, B. Faubert, Q. Yuan, N. Lev-Cohain, E. Jin, J. Kim, L. Jiang, B. Ko, R. Skelton, L. Loudat, M. Wodzak, C. Klimko, E. McMillan, Y. Butt, M. Ni, D. Oliver, J. Torrealba, C. R. Malloy, K. Kernstine, R. E. Lenkinski, R. J. DeBerardinis, Metabolic heterogeneity in human lung tumors. Cell 164, 681–694 (2016). 26853473 10.1016/j.cell.2015.12.034 PMC4752889

[14] R. C. Sun, T. W. Fan, P. Deng, R. M. Higashi, A. N. Lane, A. T. Le, T. L. Scott, Q. Sun, M. O. Warmoes, Y. Yang, Noninvasive liquid diet delivery of stable isotopes into mouse models for deep metabolic network tracing. Nat. Commun. 8, 1646 (2017). 29158483 10.1038/s41467-017-01518-z PMC5696342

[15] W. Lu, R. Miao, S. Hu, J. Liu, F. Jin, R. X. Zhang, Microsurgical skills of establishing permanent jugular vein cannulation in rats for serial blood sampling of orally administered drug. J. Vis. Exp. 178, e63167 (2021). 10.3791/63167 34978300

[16] K. Schleimer, J. Grommes, A. Greiner, H. Jalaie, J. Kalder, S. Langer, T. A. Koeppel, M. Jacobs, M. Kokozidou, Training a sophisticated microsurgical technique: Interposition of external jugular vein graft in the common carotid artery in rats. J. Vis. Exp. 69, e4124 (2012). 10.3791/4124 PMC3520580 23168988

[17] R. Makaryus, H. Lee, M. Yu, S. Zhang, S. D. Smith, M. Rebecchi, P. S. Glass, H. Benveniste, The metabolomic profile during isoflurane anesthesia differs from propofol anesthesia in the live rodent brain. J. Cereb. Blood Flow Metab. 31, 1432–1442 (2011). 21266982 10.1038/jcbfm.2011.1 PMC3130322

[18] W. W. Yang, A. W. Chen, H. Lee, H. Li, J. G. Lee, Y. Li, W. B. Shen, Isoflurane and surgical stress disrupt fatty acid and carbon metabolism, leading to cardiomyopathy in aged mice. Cells 15, 237 (2026). 41677604 10.3390/cells15030237 PMC12896423

[19] A. G. Baer, A. K. Bourdon, J. M. Price, S. R. Campagna, D. A. Jacobson, H. A. Baghdoyan, R. Lydic, Isoflurane anesthesia disrupts the cortical metabolome. J. Neurophysiol. 124, 2012–2021 (2020). 33112692 10.1152/jn.00375.2020 PMC7814899

[20] J. Stokes, A. Freed, R. Bornstein, K. N. Su, J. Snell, A. Pan, G. X. Sun, K. Y. Park, S. Jung, H. Worstman, B. M. Johnson, P. G. Morgan, M. M. Sedensky, S. C. Johnson, Mechanisms underlying neonate-specific metabolic effects of volatile anesthetics. eLife 10, e65400 (2021). 34254587 10.7554/eLife.65400 PMC8291971

[21] S. J. Yoon, J. A. Combs, A. Falzone, N. Prieto-Farigua, S. Caldwell, H. D. Ackerman, E. R. Flores, G. M. DeNicola, Comprehensive metabolic tracing reveals the origin and catabolism of cysteine in mammalian tissues and tumors. Cancer Res. 83, 1426–1442 (2023). 36862034 10.1158/0008-5472.CAN-22-3000 PMC10152234

[22] M. Grima-Reyes, A. Martinez-Turtos, I. Abramovich, E. Gottlieb, J. Chiche, J. E. Ricci, Physiological impact of in vivo stable isotope tracing on cancer metabolism. Mol. Metab. 53, 101294 (2021). 34256164 10.1016/j.molmet.2021.101294 PMC8358691

[23] C. Jang, S. Hui, W. Lu, A. J. Cowan, R. J. Morscher, G. Lee, W. Liu, G. J. Tesz, M. J. Birnbaum, J. D. Rabinowitz, The small intestine converts dietary fructose into glucose and organic acids. Cell Metab. 27, 351–361.e3 (2018). 29414685 10.1016/j.cmet.2017.12.016 PMC6032988

[24] B. Yuan, W. Doxsey, O. Tok, Y. Y. Kwon, Y. Liang, K. E. Inouye, G. S. Hotamisligil, S. Hui, An organism-level quantitative flux model of energy metabolism in mice. Cell Metab. 37, 1012–1023.e6 (2025). 39983714 10.1016/j.cmet.2025.01.008 PMC11964847

[25] A. Miller, E. M. York, S. A. Stopka, J. R. Martinez-Francois, M. A. Hossain, G. Baquer, M. S. Regan, N. Y. R. Agar, G. Yellen, Spatially resolved metabolomics and isotope tracing reveal dynamic metabolic responses of dentate granule neurons with acute stimulation. Nat. Metab. 5, 1820–1835 (2023). 37798473 10.1038/s42255-023-00890-z PMC10626993

[26] S. M. Davidson, T. Papagiannakopoulos, B. A. Olenchock, J. E. Heyman, M. A. Keibler, A. Luengo, M. R. Bauer, A. K. Jha, J. P. O’Brien, K. A. Pierce, D. Y. Gui, L. B. Sullivan, T. M. Wasylenko, L. Subbaraj, C. R. Chin, G. Stephanopolous, B. T. Mott, T. Jacks, C. B. Clish, M. G. Vander Heiden, Environment impacts the metabolic dependencies of Ras-driven non-small cell lung cancer. Cell Metab. 23, 517–528 (2016). 26853747 10.1016/j.cmet.2016.01.007 PMC4785096

[27] P. I. Zimin, C. B. Woods, E. B. Kayser, J. M. Ramirez, P. G. Morgan, M. M. Sedensky, Isoflurane disrupts excitatory neurotransmitter dynamics via inhibition of mitochondrial complex I. Br. J. Anaesth. 120, 1019–1032 (2018). 29661379 10.1016/j.bja.2018.01.036 PMC6200108

[28] S. Jung, P. I. Zimin, C. B. Woods, E. B. Kayser, D. Haddad, C. R. Reczek, K. Nakamura, J. M. Ramirez, M. M. Sedensky, P. G. Morgan, Isoflurane inhibition of endocytosis is an anesthetic mechanism of action. Curr. Biol. 32, 3016–3032.e3 (2022). 35688155 10.1016/j.cub.2022.05.037 PMC9329204

[29] M. D. Neinast, C. Jang, S. Hui, D. S. Murashige, Q. Chu, R. J. Morscher, X. Li, L. Zhan, E. White, T. G. Anthony, J. D. Rabinowitz, Z. Arany, Quantitative analysis of the whole-body metabolic fate of branched-chain amino acids. Cell Metab. 29, 417–429.e4 (2019). 30449684 10.1016/j.cmet.2018.10.013 PMC6365191

[30] M. C. Blair, M. D. Neinast, Z. Arany, Whole-body metabolic fate of branched-chain amino acids. Biochem. J. 478, 765–776 (2021). 33626142 10.1042/BCJ20200686 PMC9183206

[31] M. Arnold, W. Langhans, Effects of anesthesia and blood sampling techniques on plasma metabolites and corticosterone in the rat. Physiol. Behav. 99, 592–598 (2010). 20152845 10.1016/j.physbeh.2010.01.021

[32] E. D. Kharasch, D. C. Hankins, K. Cox, Clinical isoflurane metabolism by cytochrome P450 2E1. Anesthesiology 90, 766–771 (1999). 10078678 10.1097/00000542-199903000-00019

[33] F. F. Horber, S. Krayer, J. Miles, P. Cryer, K. Rehder, M. W. Haymond, Isoflurane and whole body leucine, glucose, and fatty acid metabolism in dogs. Anesthesiology 73, 82–92 (1990). 2360744 10.1097/00000542-199007000-00013

[34] Y. Ye, X. Fu, J. Wang, J. Fang, A. Low-Stress, Long-duration stable tail vein catheterization and precise drug delivery protocol for awake, freely moving mice. Bio. Protoc. 16, e5585 (2026). 10.21769/BioProtoc.5585 PMC12887871 41675990

[35] D. H. Tran, D. Kim, R. Kesavan, H. Brown, T. Dey, M. H. Soflaee, H. S. Vu, A. Tasdogan, J. Guo, D. Bezwada, H. Al Saad, F. Cai, A. Solmonson, H. Rion, R. Chabatya, S. Merchant, N. J. Manales, V. T. Tcheuyap, M. Mulkey, T. P. Mathews, J. Brugarolas, S. J. Morrison, H. Zhu, R. J. DeBerardinis, G. Hoxhaj, De novo and salvage purine synthesis pathways across tissues and tumors. Cell 187, 3602–3618.e20 (2024). 38823389 10.1016/j.cell.2024.05.011 PMC11246224

[36] S. Hui, J. M. Ghergurovich, R. J. Morscher, C. Jang, X. Teng, W. Lu, L. A. Esparza, T. Reya, Z. Le, J. Yanxiang Guo, E. White, J. D. Rabinowitz, Glucose feeds the TCA cycle via circulating lactate. Nature 551, 115–118 (2017). 29045397 10.1038/nature24057 PMC5898814

[37] M. F. Clasquin, E. Melamud, J. D. Rabinowitz, LC-MS data processing with MAVEN: A metabolomic analysis and visualization engine. Curr. Protoc. Bioinformatics 37, 14.11.11–14.11.23 (2012). 10.1002/0471250953.bi1411s37 PMC4055029 22389014

