# Supplementary material for: A simple, anesthesia-free infusion technique for in vivo metabolic tracing
Source: Sci Adv. 2026 Sep 11;12(37):eaeg1157. doi: 10.1126/sciadv.aeg1157 (PMC13564915; doi:10.1126/sciadv.aeg1157)
Supplement: Supplementary file 1 [file sciadv.aeg1157_sm.pdf]

Science Advances
AAAS

Supplementary Materials for

A simple, anesthesia-free infusion technique for in vivo metabolic tracing

Yumi Kim *et al.*

Corresponding author: Gina M. DeNicola, gina.denicola@moffitt.org

Sci. Adv. **12**, eaeg1157 (2026)
DOI: 10.1126/sciadv.aeg1157

The PDF file includes:

Figs. S1 to S4
Legends for movies S1 to S4

Other Supplementary Material for this manuscript includes the following:

Movies S1 to S4

Supplementary Materials

Supplementary Figures

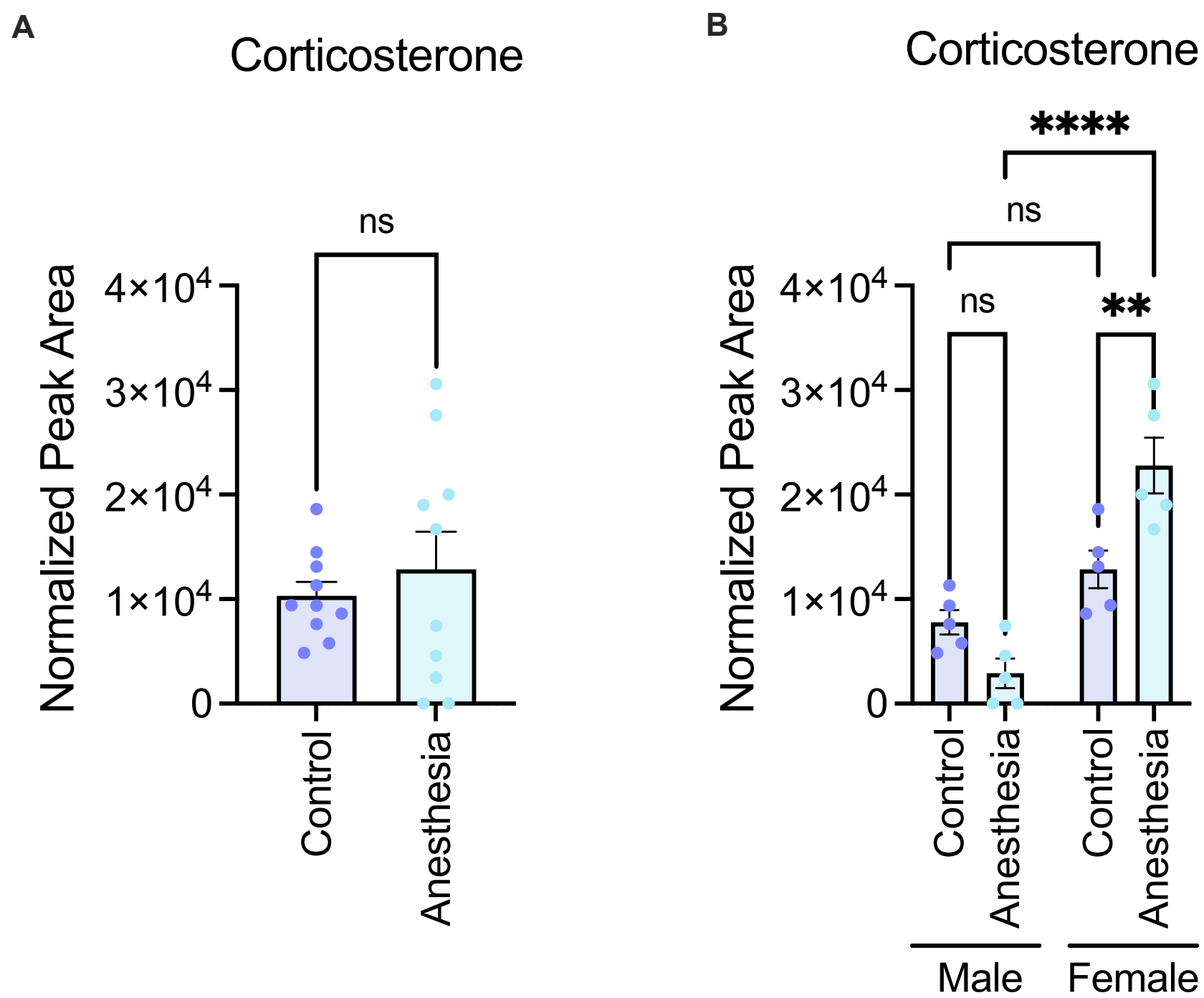

Supplementary Figure 1. Serum corticosterone levels following 2 h isoflurane anesthesia. Mice were subjected to either isoflurane anesthesia or anesthesia-free control conditions for 2 h prior to serum collection, corresponding to the experimental design shown in Figure 1A. Serum corticosterone abundance was measured by LC-MS analysis. (**A**) Total serum corticosterone abundance in anesthesia-free control and isoflurane anesthesia groups. (**B**) Sex-stratified analysis of serum corticosterone abundance in male and female mice following isoflurane anesthesia compared with anesthesia-free controls. Data are presented as mean ± SEM (n = 10 mice per group; 5 males and 5 females), with each dot representing an individual mouse. tatistical significance was determined using two-way ANOVA followed by Fisher's LSD multiple comparisons test. **P < 0.01; ****P < 0.0001; ns, not significant.

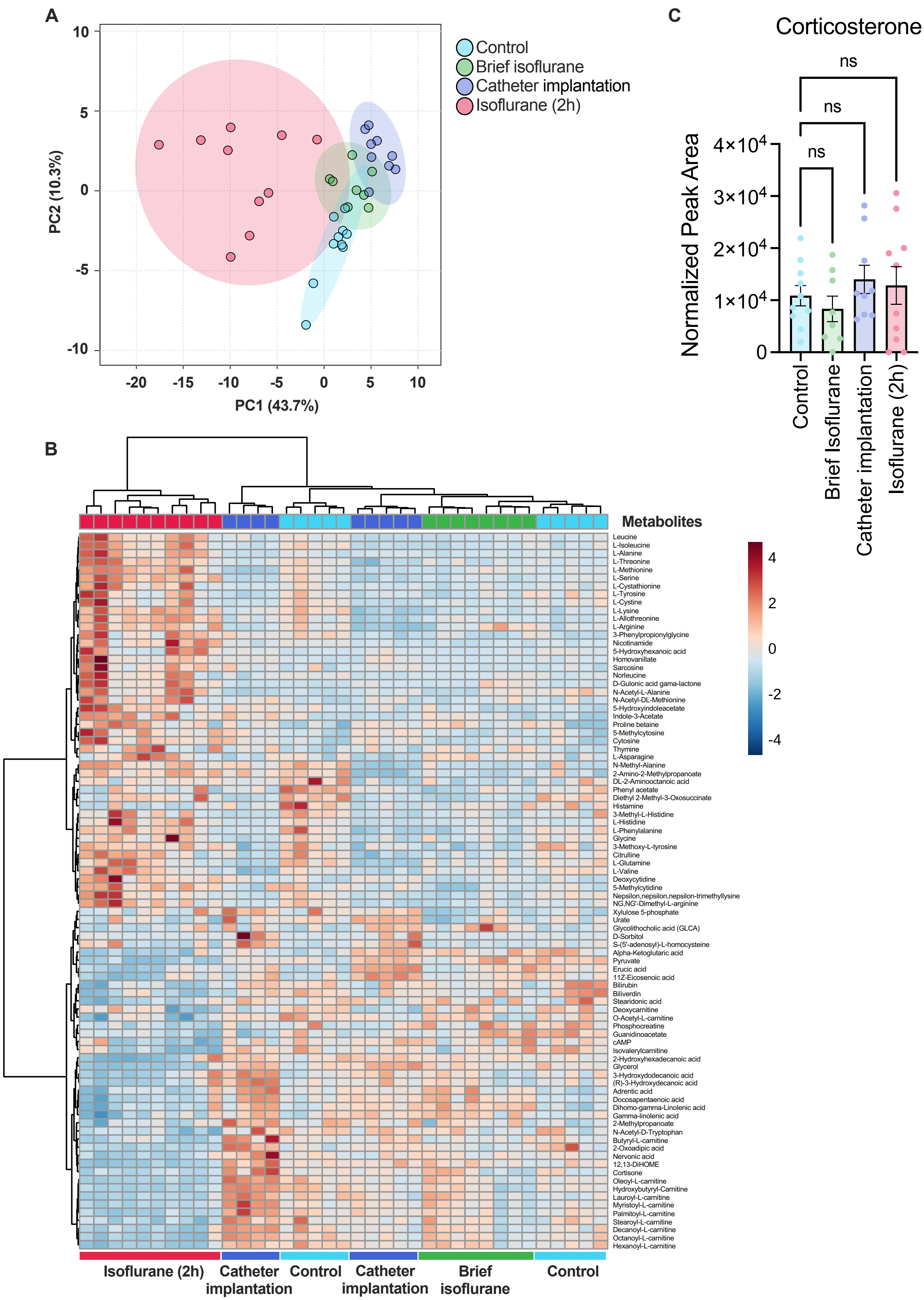

A
PC2 (10.3%)
PC1 (43.7%)
10
5
0
-5
-10
-20
-15
-10
-5
0
5
10
Control
Brief isoflurane
Catheter implantation
Isoflurane (2h)
C
Corticosterone
Normalized Peak Area
4×10^4
3×10^4
2×10^4
1×10^4
0
ns
ns
ns
Control
Brief Isoflurane
Catheter implantation
Isoflurane (2h)
B
Metabolites
4
2
0
-2
-4
Leucine
L-Isoleucine
L-Alanine
L-Threonine
L-Methionine
L-Serine
L-Cystathionine
L-Tyrosine
L-Cystine
L-Lysine
L-Allothreonine
L-Arginine
3-Phenylpropionylglycine
Nicotinamide
5-Hydroxyhexanoic acid
Homovanillate
Sarcosine
Norleucine
D-Gulonic acid gama-lactone
N-Acetyl-L-Alanine
N-Acetyl-DL-Methionine
5-Hydroxyindoleacetate
Indole-3-Acetate
Proline betaine
5-Methylcytosine
Cytosine
Thymine
L-Asparagine
N-Methyl-Alanine
2-Amino-2-Methylpropanoate
DL-2-Aminooctanoic acid
Phenyl acetate
Diethyl 2-Methyl-3-Oxosuccinate
Histamine
3-Methyl-L-Histidine
L-Histidine
L-Phenylalanine
Glycine
3-Methoxy-L-tyrosine
Citrulline
L-Glutamine
L-Valine
Deoxycytidine
5-Methylcytidine
Nepsilon,nepsilon,nepsilon-trimethyllysine
NG,NG'-Dimethyl-L-arginine
Xylulose 5-phosphate
Urate
Glycolithocholic acid (GLCA)
D-Sorbitol
S-(5'-adenosyl)-L-homocysteine
Alpha-Ketoglutaric acid
Pyruvate
Erucic acid
11Z-Eicosenoic acid
Bilirubin
Biliverdin
Stearidonic acid
Deoxycarnitine
O-Acetyl-L-carnitine
Phosphocreatine
Guanidinoacetate
cAMP
Isovalerylcarnitine
2-Hydroxyhexadecanoic acid
Glycerol
3-Hydroxydodecanoic acid
(R)-3-Hydroxydecanoic acid
Adrentic acid
Docosapentaenoic acid
Dihomo-gamma-Linolenic acid
Gamma-linolenic acid
2-Methylpropanoate
N-Acetyl-D-Tryptophan
Butyryl-L-carnitine
2-Oxoadipic acid
Nervonic acid
12,13-DiHOME
Cortisone
Oleoyl-L-carnitine
Hydroxybutyryl-Carnitine
Lauroyl-L-carnitine
Myristoyl-L-carnitine
Palmitoyl-L-carnitine
Stearoyl-L-carnitine
Decanoyl-L-carnitine
Octanoyl-L-carnitine
Hexanoyl-L-carnitine
Isoflurane (2h)
Catheter implantation
Control
Catheter implantation
Brief isoflurane
Control

Supplementary Figure 2. Brief isoflurane exposure and catheter setup do not induce persistent metabolomic alterations. Mice were assigned to four groups: handled control, brief isoflurane (2% isoflurane for 10 min), catheter implantation (tail-vein catheterization under 2% isoflurane anesthesia for 10 min), or isoflurane (2h; prolonged isoflurane exposure). Serum samples were collected either 1 h after recovery or immediately following prolonged anesthesia exposure. (**A**) Principal component analysis (PCA) of serum metabolomic profiles across experimental groups. (**B**) Hierarchical clustering heatmap of significantly altered serum metabolites across experimental groups. Heatmap values represent normalized metabolite abundance (*Z*-score). Metabolites passing a false discovery rate (FDR) threshold of 0.01 by one-way ANOVA were included. Data are from $n = 10$ for control, $n = 8$ for brief isoflurane, $n = 9$ for catheter implantation, and $n = 10$ for isoflurane (2h). (**C**) Total serum corticosterone abundance across experimental groups. Data are presented as mean ± SEM (control: $n = 10$; brief isoflurane: $n = 8$; catheter implantation: $n = 9$; isoflurane (2h): $n = 10$) with each dot representing an individual mouse. Statistical significance was determined using one-way ANOVA followed by Dunnett's multiple comparisons test. ns, not significant.

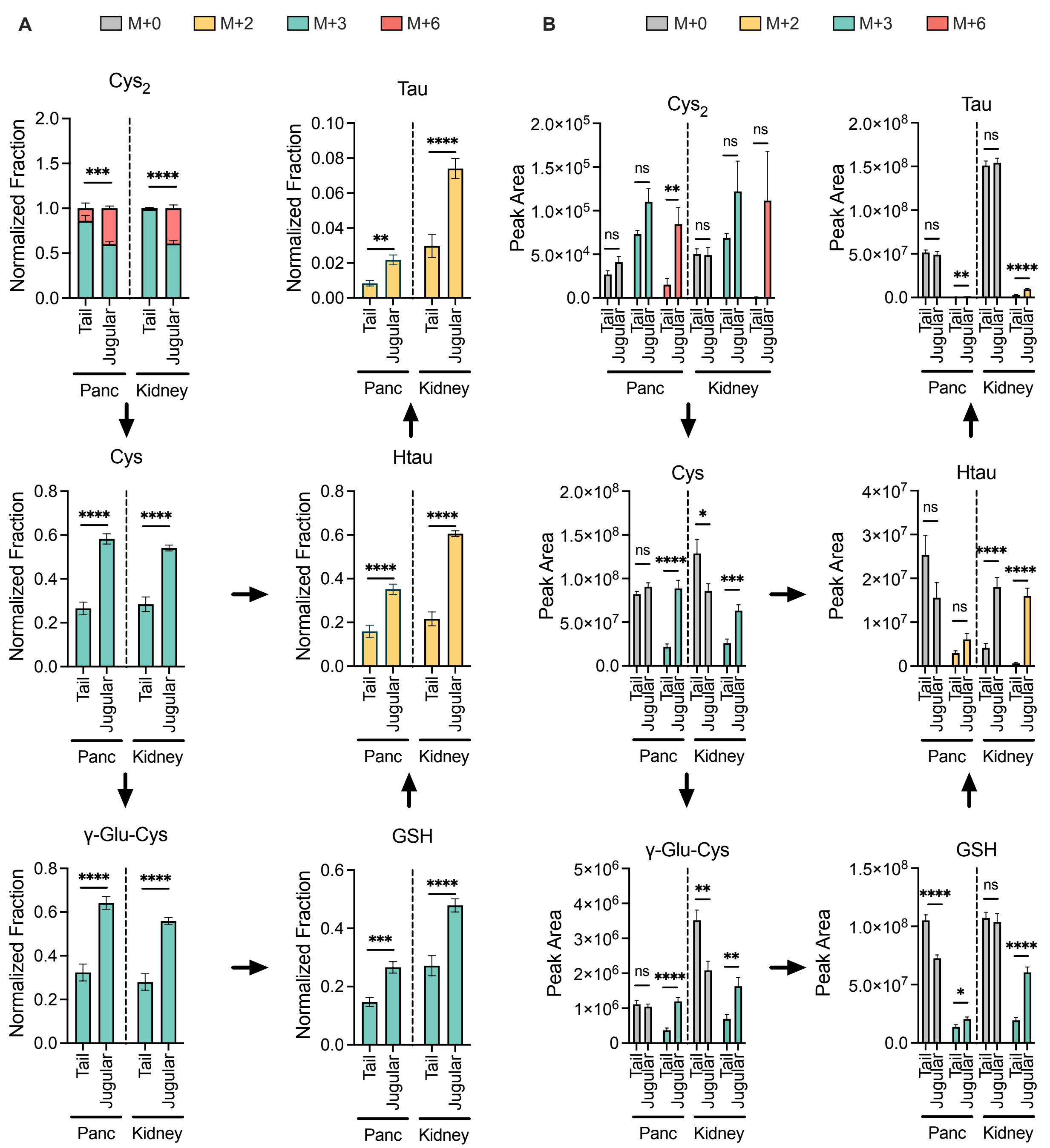

Supplementary Figure 3. Normalized isotopologue enrichment and metabolite pool size analysis of cystine-derived metabolites following jugular or tail-vein U-$^{13}C_6$-cystine infusion. (**A**) Isotopologue fractions of cystine-derived metabolites normalized to the corresponding tissue ^{13}C-cystine enrichment in pancreas and kidney. (**B**) Absolute LC-MS peak areas for unlabeled (M+0) and labeled (M+2, M+3, and M+6) isotopologues of cystine and downstream sulfur metabolites in pancreas and kidney following jugular or tail-vein infusion. Data are presented as mean ± SEM (*n* = 12 for jugular and *n* = 9 for tail vein groups). Statistical

significance was determined using a two-tailed unpaired Student's *t*-test. *$P < 0.05$; **$P < 0.01$; ***$P < 0.001$; ****$P < 0.0001$; ns, not significant.

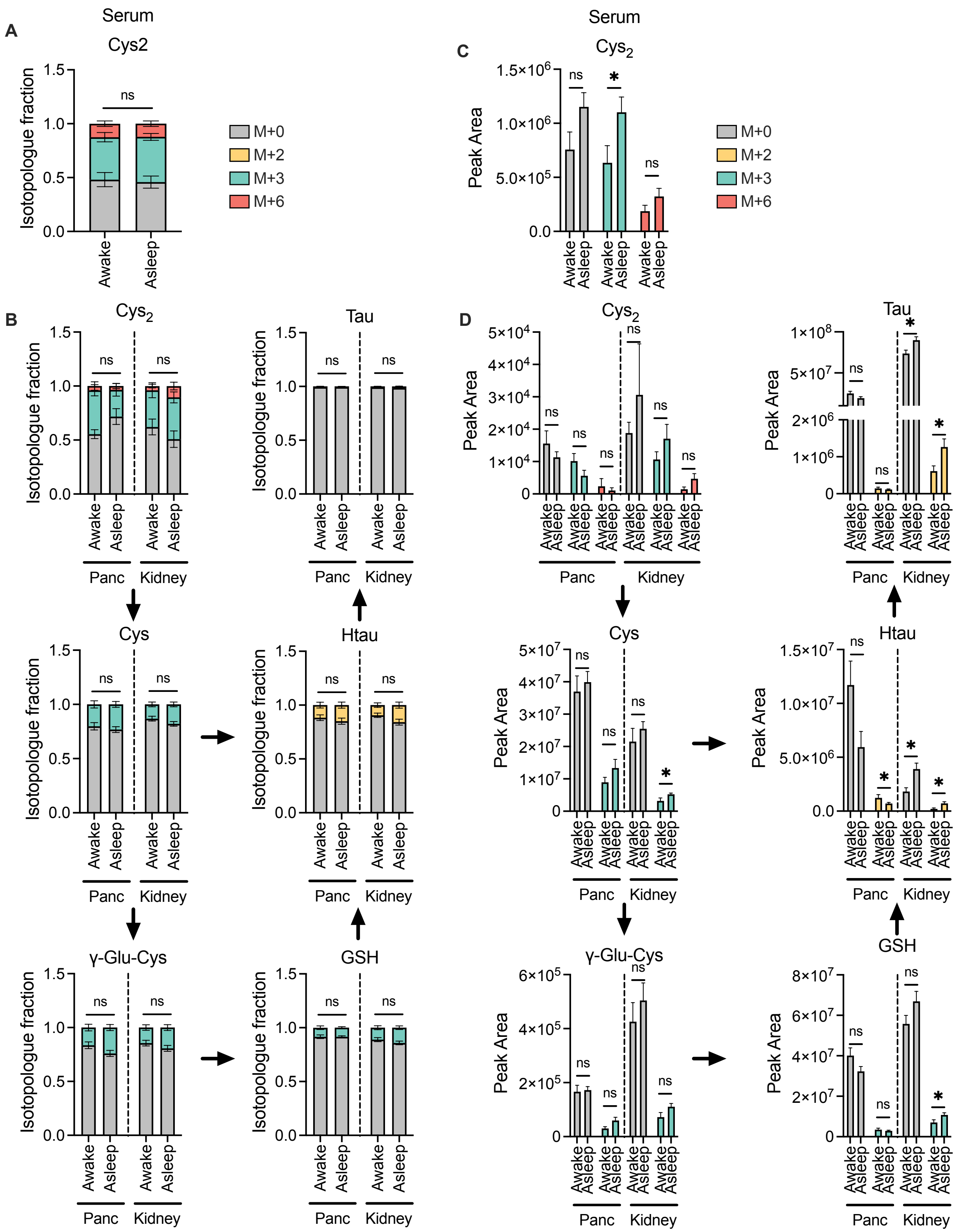

Supplementary Figure 4. Comparison of awake and anesthetized tail-vein U-$^{13}C_6$-cystine infusion. Mice received continuous U-$^{13}C_6$-cystine infusion via tail-vein catheterization (10 mg/mL,

120 μL/h) for 4 h under awake (Awake) or isoflurane-anesthetized conditions (Asleep). (**A**) Serum cystine isotopologue distributions following awake or isoflurane-anesthetized tail-vein infusion. (**B**) Isotopologue distributions of cystine and cystine-derived metabolites (cysteine, γ-glutamylcysteine, glutathione, hypotaurine, and taurine) in pancreas and kidney. (**C**) Total serum cystine pool sizes for M+0, M+3, and M+6 cystine isotopologues following awake or isoflurane-anesthetized tail-vein infusion. (**D**) Absolute LC-MS peak areas for unlabeled (M+0) and labeled (M+2, M+3, and M+6) cystine and downstream sulfur metabolite isotopologues in pancreas and kidney. Data are presented as mean ± SEM (n = 7 for awake and n = 9 for asleep groups). Statistical significance was determined using a two-tailed unpaired Student's t-test. *$P < 0.05$; **$P < 0.01$; ***$P < 0.001$; ****$P < 0.0001$; ns, not significant.

Supplementary Movies

Supplementary Movie 1. Normal home-cage behavior in a healthy mouse. This video shows a healthy C57BL/6NJ mouse (12 weeks old) in a standard home-cage environment without undergoing any experimental procedure. The mouse displays normal spontaneous movement, grooming, and exploratory behaviors.

Supplementary Movie 2, 3, and 4. Tail-vein catheter infusion in awake, freely moving mice. These videos show healthy C57BL/6NJ mice (12 weeks old) after tail-vein catheter placement, recovery from brief isoflurane anesthesia, and transfer to the infusion cage. During continuous stable isotope tracer infusion, mice remain awake and freely moving, displaying spontaneous movement, grooming, and exploratory behaviors throughout the infusion period.
